# Updates on Caries Management in the Primary and Permanent Dentition

**DOI:** 10.3390/medicina61020316

**Published:** 2025-02-11

**Authors:** Julian Schmoeckel

**Affiliations:** Department of Pediatric Dentistry, University Medicine Greifswald, 17489 Greifswald, Germany; julian.schmoeckel@uni-greifswald.de

Caries is still one of the most prevalent diseases affecting children and adults worldwide [1]. Reducing its prevalence and improving the quality of dental care, along with the oral-health-related quality of life, should, therefore, be a major goal. 

The aim of this Special Issue was to present innovative approaches, high-quality clinical, epidemiological, and dental public health research, or translational research on several aspects of the management of dental caries (preventive/non-invasive, minimally invasive, operative approaches) in children and adults.

First of all, I would like to emphasise that this Special Issue contains a variety of study types, reflecting the different options in research methodology: meta-analysis, systematic reviews, epidemiological research, laboratory studies, retrospective and prospective clinical studies, RCTs, and innovative case series. Furthermore, the Special Issue includes articles on the management of caries in primary teeth and permanent teeth, as well as root caries. This demonstrates the different approaches and perspectives in cariology.

I am delighted that this completed Special Issue contains interesting innovations relating to this topic. In the following, I would like to summarise and highlight specific aspects of the contents of this Special Issue and give my personal outlook for the future.

An epidemiological study on oral health in children in Burundi showed a significant level of caries experience and a very low treatment index, highlighting that caries treatment does not reach the children [2]. This underlines the importance of improvements in prevention and easily assessable and inexpensive management options.

The number of papers in this Special Issue dealing with silver fluoride (SDF) products shows that this is a hot topic and should not be seen as an unsatisfying compromise for so-called “third-world countries” but as a viable alternative or synergistic option for caries management at any stage and on any tooth surface in any patient worldwide. 

To my knowledge, two of the published papers [3,4] are related to award-winning presentations at the ORCA Congress 2023 [3] and 2024 [5]. Interestingly, both deal with the use of silver fluoride: (1) in primary teeth, which may eventually dramatically reduce the need for dental general anaesthesia [6], and (2) for young permanent teeth, providing the first pilot data of a reported innovative approach to managing non-cavitated proximal caries [5]. This provides new insights into the potential of silver diamine fluoride (SDF) for treatment of early childhood caries (ECC) [7], as well as options for the management of proximal caries that go beyond the findings from a recent systematic review and meta-analysis [8]. In addition, a double cohort study comparing SDF and 5% fluoride varnish showed that both materials reduced hypersensitivity in children with ECC, but that the potential for caries arrest was much higher with SDF [9].

A laboratory study has shown that an individual’s diet may influence the colour stability and microhardness of composite restorations, which are probably one of the most common treatment approaches for caries worldwide [10].

A long-term split-mouth clinical study from Lithuania compared the survival rate of resin-based and GIC-based fissure sealants and showed that their effectiveness in preventing fissure caries in permanent second molars did not differ significantly over a 10-year follow-up period [11].

A meta-analysis of RCTs on the selective caries removal technique in permanent teeth also confirms, in line with another earlier consensus from ORCA [12,13], that selective caries removal is a less invasive approach to pulp tissue perseveration compared with conventional “complete caries removal” techniques [14].

A fairly wide-ranging systematic review on the cost-effectiveness of ECC treatment shows that socio-economic, cultural, and ethnic differences need to be taken into account [15]. This is particularly important in populations with a high prevalence of ECC and with a highly polarised distribution of ECC, in order to implement the most cost-effective approaches.

The direction of minimally invasive dentistry is also favoured by the authors of a study on root caries [16], which shows that the progression of initial active root carious lesions using GICs may be an interesting and promising approach.

Last but not least, a study on decision making for caries management in children shows that this part is much more complex than a tooth-level decision. Consequently, there is a need for simple and effective treatments, while unfortunately, there is still a tendency for too invasive or unrecommended treatment suggestions. The main challenge is to always make the correct diagnosis based on appropriate examinations (clinical, radiographic, etc.). Perhaps future innovations in artificial intelligence will assist dentists in facilitating diagnosis, and robots will be able to perform the necessary restorations or procedures.

I am confident that in the future, with continued research and development of novel treatment strategies, further reductions in the caries prevalence in all age groups worldwide will be achieved. Personalised medicine, multimodal and interdisciplinary management, and digital innovations can further transform the way in which we approach the field of cariology, both clinically and scientifically. Several aspects in the field of caries management have been addressed in research, but there are still relevant gaps, mainly in the areas of research on cost-effectiveness and the translation of knowledge into everyday dental practice. These advances will bring us closer to our goal of improving the oral-health-related quality of life of millions of children, adolescents, adults, and seniors.
